# The effect of community-level smoke-free ordinances on smoking rates in men based on Community Health Surveys

**DOI:** 10.4178/epih/e2014037

**Published:** 2014-12-26

**Authors:** Hye Ah Lee, Hyesook Park, Ho Kim, Kyunghee Jung-Choi

**Affiliations:** 1 Department of Preventive Medicine, Ewha Womans University School of Medicine, Seoul, Korea; 2 Biostatistics and Epidemiology, Graduate School of Public Health, Seoul National University, Seoul, Korea

**Keywords:** Smoke-free ordinance, Ecological study, Smoking, South Korea

## Abstract

**OBJECTIVES:**

As one of smoke-free policies, communities have established the smoke-free ordinances since August 2010. Thus, this study aimed to evaluate the effects of community-level smoke-free ordinances (SFO) on smoking rates in men using multiyear Community Health Survey (CHS) data.

**METHODS:**

Data on community-level SFO were collected from a website on Enhanced Local Laws and Regulation Information System. Regional smoking-related data were obtained from CHS data from 2008 to 2012 and the age-standardized rates of current smoking in men, attempts to quit smoking, and smoke-free campaign experiences including the mean number of cigarettes smoked (smoking amount) were calculated. Repeated measures analysis of variance was performed to evaluate the effects of regional implementation of SFO and the duration on change of smoking rates.

**RESULTS:**

Overall current smoking rates and daily mean cigarettes smoked were lower in community where SFO had been implemented compared to those without implementation, and there was a significant difference in smoking rates between 2010 and 2008. Cross-sectional analysis of the effects of regional SFO revealed clear difference in rate of current smoking, but longitudinal analysis showed no significant differences. Stratifying by age groups, however, showed that groups less than 30 years of age had low smoking rates in community with ordinance compared to those without SFO since 2010. Yearly surveys measuring the number of cigarettes smoked, attempts to quit smoking, and experiences of smoke-free campaigns showed regional differences in the duration of implementation, but these differences were not significant in longitudinal analysis. Furthermore, there was a difference in regional socioeconomic characteristics between community with and without SFO implementation.

**CONCLUSIONS:**

For effective smoking control, it is necessary to evaluate current policies and develop indices to evaluate the practical implementation of ordinances. As more communities to pass the SFO, long-term observation and assessments required.

## INTRODUCTION

According to a 2013 smoking-related report from the World Health Organization, 6 million people annually die due to smoking and this number is predicted to increase to approximately 8 million by 2030 [1]. Cigarette smoke contains around 250 harmful chemical substances, 69 of them can cause cancer, so that the International Agency for Research on Cancer has classified cigarette and cigarette smoke as group 1 carcinogens [2,3]. Smoking and exposure to cigarette smoke are associated with health risks such as the onset of diseases including various cancers as well as cardiovascular and respiratory diseases. In addition, exposure of pregnant women and infants to indirect cigarette smoke has unfavorable effects such as premature birth, sudden infant death syndrome, and asthma [2,3]. A study reported about 46,000 deaths in South Korea in 2003 due to smoking, and smoking was attributed to 30.8% of deaths in men [4]. Also, the Ministry of Health and Welfare [5] estimated the economic burden due to labor loss from early death and diseases induced by smoking to be about 5.6 trillion Korean won (KRW) in 2007. For that reasons, constant efforts to decrease smoking rates by establishing the smoke-free policies have been made in South Korea and worldwide.

In South Korea, smoke-free policies started with designation of smoke-free zones by the National Health Promotion Act in 1995, and these smoke-free zones in public facilities were expanded in 1999, 2003, 2006, and 2012. In addition, a cigarette tax increase in 2004, a tobacco packing warning message in 2007, abolition of military duty-free cigarettes in 2009-2010, and restriction of advertisement of certain types of cigarettes in December 2011 have been implemented [5]. These changes and policies have resulted in a reduced smoking rate in South Korea, from a mean smoking rate of 66.3% among Korean males in 1998 to 40% range after 2007. However, this rate is still higher than the mean male smoking rate reported by the Organisation for Economic Cooperation and Development in 2013 (25.94%) [6], thus it suggesting a need for more efforts for reducing the smoking rates.

While smoking is considered a behavior driven by personal preference, smoking rates are also affected by regional characteristics including residence [7,8], poverty [9], deprivation indices [10], and accessibility to harmful environments [11]. Local governments have established smoke-free ordinance (SFO) since August 2010 to encourage smoking cessation and reduce indirect smoking exposure for non-smokers by creating smoke-free environments. Since then, many communities have announced their plans to establish ordinances regarding designation of smoke-free zones, smoking controls, and fines for smoking in smoke-free zones.

Several studies have reported that legal regulation of smoking indoors and in public places resulted in reduced smoking and indirect smoking exposure rates [12,13], acute cardiovascular disease (approximately 13% reduction) [14], and respiratory disease [15]. In South Korea, however, there remains a lack of studies that evaluate the effects of regulation for smoke-free, including implementation of smoke-free policies, restriction of cigarette accessibility, and expansion of smoke-free zones [16], thus further studies are necessary for effective smoking control.

Therefore, this study aimed to investigate the changes in smoking related indices by using Community Health Survey (CHS) data from 2008 to 2012 in order to assess the effects of community-level SFO.

## MATERIALS AND METHODS

Based on the administrative classification coding included in CHSs data, all analysis conducted at the community-level. Some data were missing owing to administrative district changes. As local government ordinances were legislated in 2010, regional characteristics and regional data for ordinance were organized by focusing on 251 community that were repeatedly sampled from 2010 to 2012. However, analysis of the repeatedly measured data also included data from 2008; therefore, the analysis was performed on 241 community except for those with missing data due to administrative district changes such as Cheonan city and Yeongi county in Chungnam province, Jeonju city in Jeonbuk province, and Changwon city in Gyeongnam province.

### Data

Data on community-level SFO legislation, including execution dates, the application dates of fine imposition, and the minimum fines were collected on the Enhanced Local Laws and Regulation Information System website (http://www.elis.go.kr). Community with SFO execution dates before December 31, 2012 were defined as ordinance-implementing and others defined as non-ordinance implementing community. The duration of ordinance implementation were calculated from December 31, 2012, and then categorized as 0-3 months, 4-6 months, 7-11 months, or more than 12 months.

This study analyzed 2008 to 2012 CHS data accessed from a website (https://chs.cdc.go.kr/chs/index.do). CHS data including current smoking status, daily mean smoking amount (number of cigarettes), attempts to quit smoking within the previous year, and experiences of smoke-free campaigns, were considered outcome variables to evaluate the effect of implementation of regional ordinances. Questions about the attempts to quit smoking changed on questionnaires after 2010; therefore, the corresponding indicator included data from 2010. As smoking rates have a clear gender difference, the result indices were calculated based only on men and refer to the guidelines for analysis using CHS raw data. In order to consider factors for regional characteristics that might affect regional ordinance implementation, further data were collected from research reports from the National Statistics Office.

The proportion of regional populations with less than a high school level of education and those who were unemployed were considered as socioeconomic indices from the 2012 CHS. The unemployed population was defined as those who responded ‘no’ to the question asking if they were currently employed. In addition, regional characteristics included community-level financial independence and self-reliance ratios as well as regional deprivation indices. The financial independence ratio is an index of the finance utilization capacity of a local government with independent discretionary power, and financial self-reliance ratio is an index of the financial independence level of a local government. The number of restaurants and tobacco retail stores was also included to evaluate the accessibility of cigarettes for purchase. Data on financial self-reliance [17], financial independence rates [18], and the number of restaurants and tobacco retail stores [19] were collected from National Statistical Offices. The regional deprivation indices is calculated as the sum of eight indices, including: (1) proportion of underdeveloped residential environments, (2) proportion of elderly in the population, (3) proportion of the population with less than a high school level of education, (4) proportion of population in lower social classes based on household members, (5) proportion of households not living in an apartment, (6) proportion of households without automobiles, (7) proportion of single-person households, and (8) proportion of households with female heads of house. Higher regional deprivation indices indicates high level of deprivation risk. It values collected from research reports on ‘health promotion strategies and programmes development for health inequalities alleviation’, it performed from 2006 to 2009 [20].

### Statistical analysis

All data were calculated on a community-level basis according to the survey administrative classification codes. For direct comparisons of survey time and community for rate, direct age-standardized rates based on population size by age groups (19-29, 30-39, 40-49, 50-59, 60-69, and older than 70 years of age) in 2010 were calculated in the analyses. In some analyses, adult age groups were subcategorized into 19-29, 30-44, 45-64, and 65 or older.

Current smoking trends from 2008 to 2012 were analyzed through regression analysis, and repeated measure analysis of variance was performed to evaluate the effects of community-level SFO implementation and duration of implementation on smoking related indices such as current smoking rate, daily mean smoking amount (number of cigarettes), attempts to quit smoking within the previous year, and experiences of smoke-free campaigns. The sphericity of repeatedly measured result indices was assessed using the Mauchly test: when the sphericity test was not satisfied, p-values were adjusted using the Huynh-Feldt-Lecoultre correction.

Finally, differences in regional characteristics between communities according to SFO implementation were analyzed using t-test or Wilcoxon rank sum test. For all statistical analyses, p<0.05 was considered statistically significant using two-tailed tests and SAS version 9.3 (SAS Institute Inc., Cary, NC, USA).

## RESULTS

From 2008 to 2012, the mean smoking rate among men decreased from 47.2% to 44.8% (β=-0.57% per year), and the mean number of cigarettes smoked by daily smokers also decreased. The experience rate of smoke-free campaigns increased after 2008, but has recently decreased. Attempts to quit smoking within the previous year also increased after 2010, but decreased in 2012. Examination of annual differences by age group revealed a less than 1% decrease in smoking rates among 30-64 years of age in 2012 compared to in 2008 and 2010; the rates of those younger than 30 decreased 5.1% and 3.1%, from 2008 and 2010, and 4.6% and 2.1% in those older than 65 years of age, respectively (Table 1).

Until December 31, 2012, about 51% of community had implemented SFO by a local government, 41% of them for more than 1 year. All communities in Seoul and Ulsan had implemented the SFO by December 2012, and the implementation rate in metropolitan cities was high, at approximately 77%. Other community, however, showed low regional ordinance implementation rates, particularly Gangwon, Jeonnam, and Jeju (Appendices 1 and 2).

Analysis of regional characteristics showed that the number of restaurants and tobacco retail stores per 1,000 people was highest in Jeju and Busan. The rate of economically inactive population was high in Gwangju (25.3%), and the proportion of the population with less than high school education was highest in Jeonbuk (26.3%) (Appendix 1).

**Figure 1.**
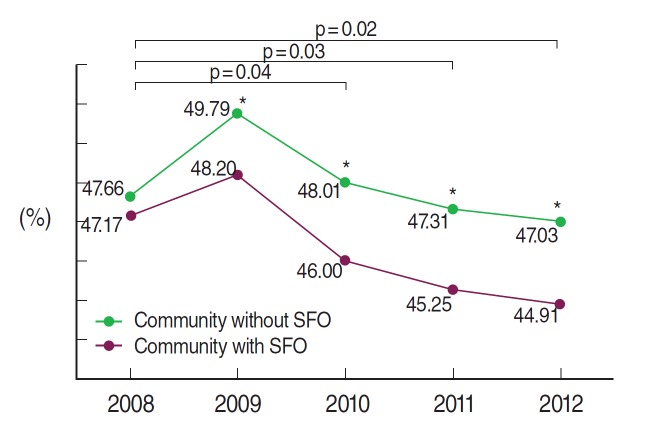
The effect of community level smoke-free ordinance (SFO) on current male smoking. It results obtained from repeated measure analysis of variance model, which includes group (community with or without SFO), time, and interaction between group and time. The p value indicates the group difference at survey year compared to baseline survey and ^*^p < 0.05 for group difference at survey year.

The overall current smoking rates in men in community that had implemented ordinances were low, with an approximately 2% difference after 2010 compared to community without implementation. Annual smoking rates according to ordinance implementation showed significant differences since 2009 (Table 2), and the degree of differences in regional smoking rates differed significantly in 2010 compared to 2008 (Figure 1). However, the decreased smoking rates over time in community with SFO implementation were only borderline significant (p_G*T_= 0.06) (Table 2). Age stratification of regional smoking rate patterns according to SFO implementation showed that smoking rates in community with implementation were lower overall, except among 30 years or less. However, this age group showed lower smoking rates in community with SFO implementations after 2010 compared to those without implementation, and the gap was even larger in 2012 (Appendix 3).

Similar to smoking rates, the daily mean smoking amount was also lower in community with SFO implementation; however, the decreased smoking amount over time in community with ordinance implementation was not statistically significant. The rates of attempts to quit smoking was higher in community with ordinance implementation, although the rates decrease in the recent year. During 2010 and 2011, however, it showed a larger increase in community with ordinance implementation (3.6%) compared to those without (2.4%). Experience rates of smoke-free campaigns were not differ significantly according to community with or without ordinance implementation (p_*G*_= 0.81). However, experience rates of smoke-free campaigns during 2010 and 2011 increased 2.7% and 5.0% in community without and with ordinance implementation, respectively. It was a statistically significant difference (Table 2).

Analysis of the current smoking rates according to duration since ordinance implementation (no SFO, less than 3 months, less than 6 months, less than 1 year, or more than 1 year) showed consistently lower current smoking rates in men in community with more than 12 months of ordinance implementation, with significant differences in rates since 2009. In cross-sectional analysis, the number of cigarettes smoked, attempts to quit smoking, and experience of smoke-free campaigns showed regional differences according to implementation duration, but these differences were not significant in longitudinal analysis (Table 3).

The differences in regional characteristics according to SFO implementation were given in Table 4. The number of restaurants and tobacco retail stores in community with ordinance implementation was 2.1 per 1,000 people, it was lower than community without implementation (3.0 per 1,000 people). The proportion of the population with less than a high school level of education was also lower in community with ordinance implementation, compared to those without (17.1% vs. 23.1%, p<0.0001). However, unemployment rates were higher in community with ordinance implementation compared to those without. In addition, deprivation indices were high in community without ordinance implementation, and both financial independence and financial self-reliance ratios were also significantly higher in community with ordinance implementation than those without (Table 4).

## DISCUSSION

This study evaluated community-level SFO as one of regional control for smoke-free, it was regional differences for timing the ordinance implementation. As a result, ordinances were implemented in more than 50% of communities by December 2012, and overall smoking rates and daily mean smoking amount were lower in community with SFO implementation than those without. In addition, smoking rates according to ordinance implementation showed a significant difference in 2010 compared to 2008. In particular, the rates of smoking cessation trial within the previous year and experience of smoke-free campaigns between 2010 and 2011 had larger increases in community with ordinance implementation compared to those without; comparison of the duration of ordinance implementation showed a greater change in community with earlier implementation of smoke-free controls, but this difference was not statistically significant.

Smoking is an avoidable risk factor for various smoking-related diseases, and has undesirable health effects on non-smokers as well as smokers exposed to cigarette smoke. For that reasons, concerted efforts have been made internationally to reduce and prevent cigarette-related health risks through establishment of smoke-free policies. Generally, smoke-free policies can be largely classified into price and non-price policies: Non-price policies include smoke-free campaigns and support for smoke-free projects. As a results of the implementation of tobacco price increases in December 2004, the 2001 smoking rate in men decreased from 60.9% (standardized rate) to 51.6% in 2005 and 45.0% in 2007 [21]. A study of analysis for ‘willingness-to-quit’ cigarette price by Shin [22] estimated the reduction of smoking rates induced by elevation of tobacco prices, concluding that about 41 and 73% of current smokers were expected to quit smoking when tobacco prices increased to 3,000 and 4,000 KRW, respectively. Some studies have reported that the rate of tobacco price increases is lower than income increases, thus cigarette consumption are not very high in economic burdens of household [5].

One example of non-price smoking policies in South Korea, smoke-free zones have been expanded since 1995, and a revision of the National Health Promotion Act in December 2012 banned smoking in public institutions and public facilities. A Cochrane systematic review the effects of legal regulations such as designation of smoke-free zones in public places, workplaces, and restaurants [13] showed a decrease of secondhand smoking exposure rate, but it could not reach the conclusion in current smoking rate. In Ireland, one year after smoking ban policies were implemented in workplaces including service businesses in March 2004, the smoking rate decreased from 29% to 26% but increased to 28% the following year [23]. In the UK, the rate of smoke cessation increased within a year after implementation of smoke-free legislation in July 2007, but this effect did not last [23].

In present study, there was a trend towards decreasing smoking rates after 2008, although this change was not great, it contributed to the reduction in age groups less than 30 and older than 65 years of age. In addition, analysis of the effects of community-level SFO using the annual survey data revealed differences between community with or without ordinance implementation at survey year, but the decreased smoking rates over time in community with ordinance implementation did not differ significantly. To consider the period of annual survey (every year from August to October), community that implemented ordinance before August 2012 showed a lower smoking rates. Similar to the findings above, although there were regional differences based on survey year, longitudinal analysis did not show differences in the smoking. Comparison of the effects of community-level SFO by age group showed that younger age groups (less than 30 years) in community with ordinance implementation had decreased smoking rates after 2010 compared to those without. A study performed in Italy on the effects of smoking regulation in young age groups reported a notable decrease in the smoking rate of 15-24 year-olds after implementation of smoke-free regulation in indoor public places [12]. Decreased smoking rates in the younger age groups in the present study would likely influence the high rate of attempts to quit smoking and experience of smoke-free campaigns. However, further studies are required to observe such a trend for longer periods of time.

Most studies on smoke-free policies evaluated rates of current smoking, indirect smoking exposure, and smoking cessation trial as an index of recognition and attitude changes among smokers. Smoke-free policies are implemented nationwide simultaneously; therefore, the rates of change before and after policy implementation are typically evaluated. In the US, two indices have been developed to assess the effects of the American Stop Smoking Intervention Study. The initial outcome index indicates the degree of state smoke-free intervention policies such as indoor air legislation and cigarette prices, and strength of tobacco control measures tobacco control resources, capacities, and program, both are correlated to state-wide decreases in smoking rates [8].

Individual smoking habits can be influenced by the surrounding environment, society, and culture [11,24]. Residence community [7,8], regional socioeconomic levels (poverty levels and unemployment rates) [9,10], and better accessibility to cigarettes are associated with individual smoking habits [11]. In a study using 2009 CHS data, regional variations including regional deprivation indices, the number of bars per 1,000 people, and the level of smoke-free education for residents were associated with smoking rate [25]. In general, regional regulations include smoke-free public relations and campaign activities in addition to the authority provision to designate smoke-free zones and impose fines for violations in smoke-free zones in order to establish smoke-free environments. In this study, financial independence and financial self-reliance ratios show that the financial capacity of communities were used to evaluate the possibility of implementing smoking cessation projects. Overall financial independence and financial self-reliance ratios were higher in community that had implemented ordinances compared to those that had not. In addition, population socioeconomic factors including the proportion of the population with less than a high school level of education and regional deprivation indices were considered regional characteristics. The analysis showed that the proportion of the population with less than a high school education in community that had implemented ordinances was around 6% lower than in community without ordinance implementation, and regional deprivation indices were also significantly lower. In addition, the number of restaurants and tobacco retail stores per 1,000 people in the community with ordinance implementation was lower than in those without (2.1 per 1,000 people vs. 3.0 per 1,000 people, p<0.0001). Unequal distribution of resources and opportunities for the regional police making can cause inequality in health [26], and regional SFO implementation also showed differences depending on regional characteristics. Although regional SFO have been continuously expanded, there are discrepancies in fine imposition and the scale of smoke-free projects in ordinance provisions; future studies will be necessary to determine the effects of these discrepancies on smoking rates.

This study only considered implementation of ordinances and it did not covered direct indices such as the number of control for smoke-free zone, the number of violations, or smoke-free budgets that could assess the practical performance of regional ordinances. Therefore, the actual effects of these ordinances may not be accurately reflected in these results. In fact, a remarkable difference on the number of fine impositions has been reported among the community with ordinance implementation. Consequently, there was required to explore indices regarding practical ordinance implementation to assess the effects of SFO. Furthermore, consistent assessments and long-term observations will be necessary. This study was limited in explanation of results due to ecological study approach and available data owing to changes in survey questions about attempts to quit smoking. As the survey were conducted annually, estimated rate for smoking related indices can be mixed and regional differences in implementation time would affect rate calculations. Nevertheless, this study offers an evaluation of the effect of community-level SFO that utilizes multiyear data. The results of this study seems to be used as preliminary data for future studies.

The objective of this study was to evaluate the effects of a community-level SFO on smoking related indices using multiyear CHS data. There were significant differences in smoking rates in community with ordinance implementation in 2010 compared to 2008, and the younger group aged 30 years or less had decreased smoking rates in community that implemented ordinances compared to those that did not. Also, there were differences in regional characteristics between community with and without ordinance implementation. Therefore, efforts should be made to avoid regional inequalities when establishing regional policies and require long-term evaluations including exploration assessable indices regarding practical ordinance implementation.

## Figures and Tables

**Table 1.** The prevalence of smoking related factors in male according to survey year

Age groups	2008		2009		2010		2011		2012	
	LSmeans	SE	LSmeans	SE	LSmeans	SE	LSmeans	SE	LSmeans	SE
Current smoker (%)										
All	47.21	0.24	48.09	0.21	46.18	0.22	45.23	0.21	44.80	0.21
19-29	46.89	0.63	47.21	0.52	44.89	0.55	43.02	0.55	41.82	0.56
30-44	56.16	0.40	57.69	0.35	56.14	0.37	55.59	0.36	55.60	0.38
45-64	44.80	0.39	45.83	0.34	43.85	0.34	43.12	0.34	42.89	0.33
≥65	28.36	0.48	27.91	0.44	25.88	0.42	24.78	0.41	23.75	0.41
Cigarettes/d (gabi)										
All	16.38	0.04	16.56	0.02	16.47	0.01	16.49	0.01	16.17	0.01
19-29	13.56	0.09	13.58	0.04	13.61	0.01	13.83	0.03	13.58	0.01
30-44	16.68	0.06	16.75	0.03	16.51	0.01	16.46	0.02	16.20	0.01
45-64	18.28	0.07	18.76	0.03	18.67	0.01	18.58	0.02	18.18	0.01
≥65	14.76	0.12	14.71	0.05	14.88	0.00	14.97	0.03	14.66	0.03
Smoking cessation trial (%)^1^										
All	-	-	-	-	29.77	0.29	33.21	0.30	27.04	0.29
19-29	-	-	-	-	35.70	0.79	41.49	0.82	34.53	0.82
30-44	-	-	-	-	31.80	0.45	35.85	0.48	28.66	0.45
45-64	-	-	-	-	26.69	0.47	28.80	0.48	23.28	0.44
≥65	-	-	-	-	22.30	0.80	23.71	0.84	20.06	0.81
Experience of smoke-free campaign (%)										
All	82.98	0.20	82.08	0.17	83.12	0.17	87.30	0.15	81.30	0.18
19-29	84.51	0.45	84.70	0.38	84.70	0.40	87.78	0.38	81.18	0.45
30-44	86.46	0.28	85.53	0.25	86.12	0.26	89.20	0.23	83.32	0.28
45-64	81.95	0.30	80.85	0.28	82.64	0.27	87.53	0.23	81.64	0.27
≥65	72.99	0.48	70.76	0.46	72.75	0.43	80.05	0.39	74.42	0.42

LSmeans, least squares means; SE, standard error.

1 Because question of smoking cessation trial changed from 2010, this table included results derived from survey which conducted in 2010, 2011, and 2012.

**Table 2.** The effect of community level smoke-free ordinance (SFO)

	Survey year					P-value
	2008	2009	2010	2011	2012	
	LSmeans	LSmeans	LSmeans	LSmeans	LSmeans	
Current smoker (%)						P_G_<0.001
Community without SFO	47.66	49.79^*^	48.01^*^	47.31^*^	47.03^*^	P_T_<0.001
Community with SFO	47.17	48.20^*^	46.00^*^	45.25^*^	44.91^*^	P_G*T_= 0.06
Cigarettes/d (gabi)						P_G_<0.001
Community without SFO	16.97^*^	17.19^*^	17.12^*^	17.05^*^	16.73^*^	P_T_<0.001
Community with SFO	16.41^*^	16.56^*^	16.32^*^	16.43^*^	16.17^*^	P_G*T_= 0.51
Smoking cessation trial (%)^1^						P_G_<0.001
Community without SFO	-	-	24.44^*^	26.71^*^	21.86^*^	P_T_<0.001
Community with SFO	-	-	29.79^*^	33.39^*^	26.98^*^	P_G*T_= 0.30
ExPerience of smoke-free camPaign (%)						P_G_= 0.81
Community without SFO	80.72	82.36	84.39	87.13	81.77	P_T_<0.001
Community with SFO	82.75	81.81	82.38	87.37^2^	81.27	P_G*T_=0.07

Communities classified into two groups according to implementation of SFO by Dec 31, 2012.

Repeated measure analysis of variance model is used for estimation. It model include group (community with or without SFO), time, and interaction between group and time.

LSmeans, least squares means; p_G_, p value for group effect; p_T_, p value for time effect; p_G*T_, p value for interaction effect between group and time.

1 Because question of smoking cessation trial changed from 2010, this table included results derived from survey which conducted in 2010, 2011, and 2012.

2 The rate differences between 2010 and 2011 by community with or without SFO was statistically significant (p=0.02).

* p<0.05 for group difference (community without/with SFO) at survey year.

**Table 3.** The effect of duration of implementation of smoke-free ordinance^1^

	Survey year					P-value
	2008	2009	2010	2011	2012	
	LSmeans	LSmeans	LSmeans	LSmeans	LSmeans	
Current smoker (%)						
Not yet	47.66	49.79^*^	48.01^*^	47.31^*^	47.03^*^	P_G_=0.002
0-3 mo	48.21	48.16^*^	46.95^*^	46.18^*^	45.74^*^	P_T_<0.001
4-6 mo	46.93	48.69^*^	46.69^*^	45.77^*^	45.18^*^	P_G*T_=0.47
7-11 mo	47.68	50.47^*^	46.80^*^	46.57^*^	46.57^*^	
≥12 mo	46.60	47.31^*^	44.96^*^	44.14^*^	43.86^*^	
Cigarettes/d (gabi)						
Not yet	16.97^*^	17.19^*^	17.12^*^	17.05^*^	16.73^*^	P_G_<0.001
0-3 mo	16.52^*^	16.56^*^	16.29^*^	16.57^*^	16.16^*^	P_T_=0.002
4-6 mo	16.54^*^	16.55^*^	16.37^*^	16.49^*^	16.16^*^	P_G*T_=0.84
7-11 mo	16.55^*^	17.19^*^	16.65^*^	16.62^*^	16.82^*^	
≥12 mo	16.26^*^	16.37^*^	16.21^*^	16.28^*^	15.97^*^	
Smoking cessation trial (%)^2^						
Not yet	-	-	24.44^*^	26.71^*^	21.86^*^	P_G_<0.001
0-3 mo	-	-	29.23^*^	30.89^*^	24.95^*^	P_G_<0.001
4-6 mo	-	-	30.93^*^	32.09^*^	28.85^*^	P_G*T_ = 0.20
7-11 mo	-	-	26.59^*^	31.04^*^	24.52^*^	
≥12 mo	-	-	30.53^*^	36.02^*^	27.90^*^	
ExPerience of smoke-free camPaign (%)						
Not yet	80.72	82.36	84.39^*^	87.13	81.77	P_G_= 0.40
0-3 mo	80.36	82.04	83.64^*^	87.55	81.58	P_T_<0.001
4-6 mo	84.66	83.44	84.69^*^	89.34	82.32	P_G*T_=0.15
7-11 mo	80.20	78.42	82.96^*^	87.89	80.27	
≥12 mo	83.88	81.97	80.52^*^	86.23	80.95	

Repeated measure analysis of variance model is used for estimation. It model includes group (not yet, 0-3 mo, 4-6 mo, 7-11 mo, ≥12 mo), time, and interaction between group and time.

Duration calculated from the enforcement date of the smoke-free ordinance to Dec 31, 2012 and then communities classified into five groups (not yet, 0-3 mo, 4-6 mo, 7-11 mo, ≥12 mo).

LSmeans, least squares means; p_G_, p value for group effect; p_T_, p value for time effect; p_G*T_, p value for interaction effect between group and time.

1 0-3 mo: Sep 2012-Dec 2012; 4-6 mo: Jun 2012-Aug 2012; 7-11 mo: Jan 2012-May 2012; ≥12 mo: earlier than Jan 2012.

2 Because question of smoking cessation trial changed from 2010, this table included results derived from survey which conducted in 2010, 2011, and 2012.

* p<0.05 for differences of duration of implementation at survey year.

**Table 4.** The differences of regional characteristics by smoke-free ordinance (SFO)

	Community without SFO	Community with SFO	P-value^1^
The rate of economically inactivity PoPulation (%)	19.97 (4.46)	21.79 (3.08)	<0.001
The number of restaurant and tobacco retail store/1,000 person	2.98 (2.00)	2.06 (1.28)	<0.001
The rate of less than a high school education level (%)	23.10 (5.48)	17.08 (6.14)	<0.001
Regional deprivation indices	0.44 (0.82)	-0.46 (0.72)	<0.001
Fiscal self-reliance ratio (%)			
2008	17.50 (12.90-25.10)	39.10 (23.50-55.20)	<0.001
2009	18.05 (12.65-24.80)	38.40 (23.80-55.20)	<0.001
2010	18.25 (12.45-25.70)	38.60 (23.10-54.40)	<0.001
2011	17.40 (12.50-24.90)	40.50 (26.40-50.60)	<0.001
2012	16.95 (12.30-26.05)	39.00 (25.50-49.10)	<0.001
Financial indePendence ratio (%)			
2008	65.95 (61.30-70.65)	70.70 (61.70-76.90)	<0.001
2009	65.30 (60.90-69.35)	69.70 (61.30-76.70)	<0.001
2010	63.00 (58.30-67.45)	67.50 (58.80-73.90)	<0.001
2011	63.40 (58.85-67.50)	66.90 (59.20-72.60)	<0.01
2012	64.20 (59.25-67.35)	66.40 (57.80-72.10)	0.01

Values are presented as mean with standard deviation or median with interquartile range for non-normal distribution.

1 p-value obtained from t-test for parametric test and Wilcoxon rank sum test for non-parametric test.

## Appendix 1. Regional characteristics in South Korea

**Table:**

Districts	No. of community	Duration of implementation of smoke-free ordinance by Dec 31, 2012^1^					No. of restaurant and tobacco retail store/1,000 person	Rate of economically inactivity population (%) inactivity population (%)	Rate of under high school education level (%)
		Not yet	0-3 mo	4-6 mo 7-1	1 mo	≥12 mo			
Seoul	25	0	0	0	0	25	1.86 (1.00)	22.80 (2.47)	13.40 (4.50)
Busan	16	8	2	2	1	3	3.95 (3.20)	24.34 (3.65)	17.32 (3.88)
Daegu	8	3	4	0	1	0	2.56 (1.77)	24.06 (2.38)	17.83 (4.13)
Incheon	10	2	3	5	0	0	2.79 (1.98)	21.50 (3.99)	17.98 (3.81)
Gwangju	5	2	1	2	0	0	2.60 (1.73)	25.31 (2.20)	14.89 (3.51)
Daejeon	5	2	3	0	0	0	1.66 (0.49)	24.79 (2.92)	16.59 (5.42)
Ulsan	5	0	0	0	0	5	1.88 (0.62)	20.24 (1.24)	16.90 (1.82)
Kyonggi	44	7	5	11	9	12	1.25 (0.41)	21.80 (2.65)	14.98 (5.08)
Gangwon	18	17	1	0	0	0	3.20 (1.14)	19.71 (4.77)	23.82 (4.40)
Chungbuk	13	9	0	2	0	2	1.82 (0.81)	19.68 (3.49)	23.00 (4.39)
Chungnam	17	13	3	1	0	0	3.38 (3.32)	18.48 (3.98)	24.68 (6.31)
Jeonbuk	15	11	0	0	1	3	2.91 (1.10)	20.74 (4.99)	26.32 (5.97)
Jeonnam	22	18	3	0	1	0	3.29 (1.14)	17.67 (3.34)	26.28 (5.06)
Gyeongbuk	24	21	2	0	1	0	2.84 (1.41)	18.86 (2.88)	24.41 (4.44)
Gyeongnam	22	9	2	1	7	3	2.48 (1.05)	20.44 (3.61)	21.80 (4.49)
Jeju	2	2	0	0	0	0	4.96 (1.50)	15.14 (5.91)	17.78 (3.95)
Total	251	124	29	24	21	53	2.51 (1.74)	20.89 (3.93)	20.05 (6.55)

1 0-3 mo: Sep. 2012-Dec. 2012; 4-6 mo: Jun. 2012-Aug. 2012; 7-11 mo: Jan. 2012-May. 2012; ≥12 mo: earlier than Jan 2012.

## Appendix 2.

The community with implementation of smoke-free ordinance by Dec 31, 2012. Black indicates the community with implementation of smoke-free ordinance by Dec 31, 2012.

## Appendix 3.

The effect of community level smoke-free ordinance (SFO) on current male smoking by age groups (A) 19-29 yr, (B) 30-44 yr, (C) 45-64 yr, and (D) more than 65 yr. It results obtained from repeated measure analysis of variance model, which includes group (community with or without SFO), age group (19-29 yr, 30-44 yr, 45-64 yr, more than 65 yr), time, and interaction between them.
