# Supplementary material for: The effect of community-level smoke-free ordinances on smoking rates in men based on Community Health Surveys
Source: Epidemiol Health. 2014 Dec 26;36:e2014037. doi: 10.4178/epih/e2014037 (PMC4371388; doi:10.4178/epih/e2014037)
Supplement: Supplementary file 1 [file epih-36-e2014037-supplementary.pdf]

원저

남성 흡연율에 대한 지역별 금연 조례 시행에 대한 효과: 지역사회건강조사 자료 이용

The effect of community level smoke-free regulations on smoking rate in male: Using Community Health Survey

이혜아, 박혜숙, 김호, 정최경희,

예방의학교실, 이화여자대학교 의학전문대학원

Hye Ah Lee[1], Hyesook Park[1], Ho Kim[2], Kyunghee Jung-Choi[1]

[1]Department of Preventive medicine, School of Medicine, Ewha Womans University,

[2]Biostatistics and Epidemiology, School of Public Health, Seoul National University, Seoul, Republic of Korea

교신저자: 이혜아

주소: (158-710)

서울시 양천구 안양천로 1071 이화여자대학교 의학전문대학원 예방의학교실

전화번호: 02) 2650 - 5753

Fax 번호: 02) 2652 - 8325

E-mail 주소: khyeah@naver.com

초록

목적: 본 연구에서는 2010년 8월부터 제정된 지방자치단체의 금연관련 조례가 금연에 미친 영향을 평가하고자 하였다.
방법: 지역 단위는 시·군·구 수준으로 자료를 수집하였으며, 지역별 금연관련 조례제정 자료는 자치법규정보 사이트로부터 수집하였다. 지역별 흡연 관련 자료는 2008년부터 2012년까지 조사된 지역사회건강조사 자료로부터 남성의 현재흡연, 평균 흡연량(개비), 금연 시도, 금연캠페인 경험에 대해 표준화율을 산출하여 이용하였다. 다년도의 흡연 관련 지표 율의 변화에 대해 지역별 조례 시행 여부 혹은 조례 시행 기간에 효과를 평가하기 위해, 반복측정 분산분석(repeated measure analysis of variance)을 이용하여 분석을 수행하였다.
결과: 전반적으로 조례 시행지역의 흡연율 및 일일 평균 흡연량은 미시행지역보다 낮았으며, 조례 시행여부에 따른 지역간의 흡연율 차이는 2008년과 비교하여 2010년부터 유의하게 차이가 있는 것으로 나타났다. 지역별 금연 조례 시행에 대한 효과에 있어서 조사시점 별로 분석하였을 때, 흡연율에 있어서 조례 시행 지역간의 뚜렷한 차이가 존재하였으나 종단적 분석을 통해 접근하였을 때 시간에 따른 흡연율 감소 폭은 조례 시행 지역간에 차이를 보이지 않았다. 그러나 연령별로 층화하여 보았을 때 30세 미만 젊은 연령군에서 2010년부터 조례 시행지역의 흡연율이 미시행지역보다 낮아지는 변화를 보였다. 이 밖에 흡연량, 금연 시도율 및 캠페인 경험률의 경우 시행 기간에 따른 지역간의 차이는 조사 시점에 따라 차이를 보였으나, 종단적으로 분석하였을 때 유의한 차이를 보이지 않았다. 또한, 조례 시행지역과 미시행지역간의 지역의 사회경제적 특성 요인에 차이가 존재하는 것으로 나타났다.
결론: 효과적인 흡연 규제를 위해, 현재 시행중인 정책에 대해 효과를 평가 할 필요가 있으며, 실질적인 조례 실행 정도를 평가할 수 있는 지표 탐색이 필요할 것으로 보인다. 또한, 지역별 조례제정이 확대되고 있음에 따라, 향후 장기적인 평가가 필요할 것으로 보인다.

주제어; Ecological study, Smoke-free regulation, South Korea, Smoking

서론

2013년 세계보건기구(WHO)의 흡연 관련 보고서에 따르면, 흡연으로 인해 매년 6백만명이 사망하며, 2030년까지 약 8백만명까지 증가 할 것으로 예측하였다[1]. 담배 연기는 약 250종의 유해한 화학물질을 포함하고 있으며, 그 중 69종은 암을 유발시킬 수 있는 물질로, 국제 암연구소(International agency for research on cancer, IARC) 에서는 담배 및 담배연기에 대해 인체발암물질(Group 1)로 분류하였다[2,3]. 흡연 및 담배연기 노출에 따른 건강위험은 각종 암 질환뿐만 아니라, 심혈관/호흡기 질환 등 다양한 질환 발생에 영향을 미치며 임산부 및 소아에게 간접적으로 담배 연기 노출 시, 조산 및 영아돌연사 증후군, 천식 유발 등 좋지 않은 건강 영향을 미치는 것으로 알려져 있다[2,3]. 국내에서 흡연으로 인한 사망자수 추정에 대한 연구로, 2003년 흡연으로 인한 사망자 수는 약 4만 6천명이며, 남성 사망의 30.8%가 흡연에 기인한 것으로 보고한 바 있다[4]. 또한, Jung et al[5]의 연구에서 흡연으로 위한 조기사망 및 질환으로 인한 노동력 손실 등에 의한 경제적 부담은 2007년 기준 약 5조 6천억원으로 추정하였다. 이와 같이 흡연에 대한 위해성 및 질병부담이 분명함에 따라 국내뿐만 아니라 국제적으로 국가 수준에서 금연관련 정책 마련을 통해 흡연율을 낮추기 위해 지속적으로 노력하고 있다.

우리나라의 경우, 1995년 국민건강증진법 제정을 통해 금연구역을 지정함으로써 본격정인 금연 정책을 시행하기 시작하여, 1999년, 2003년, 2006년, 2012년에 공중이용시설의 금역구역을 지속적으로 확대하고 있다. 이 밖에 담뱃세 인상(2004년), 발암성 물질 경고 문구 표시(2007년), 군면세 담배 폐지(2009-2010년), 품종별 담배광고 제한(2011년 12월)등을 시행하였다[6]. 국내 흡연율을 줄이고자 하는 정책 시행을 통해 국내 남성의 평균 흡연율은 1998년 66.3%에서 2007년 이후 40%대 수준을 보이고 있다. 그러나 이는 2013년 OECD 보고서에 보고한 평균 남성흡연율 보다는 높은 상태로(25.94%)[7], 지속적인 금연을 위한 대책이 필요할 것으로 보인다.

흡연은 개인의 기호에 의한 행태로 고려되지만, 거주하는 지역[8,9], 지역의 빈곤 정도[10], 지역박탈지수[11], 유해환경 접근성 [12] 등 지역 특성에 따라 차이를 보일 수 있다. 이에 지역사회에서 금연 환경 조성을 통해 흡연자에게 금연을 유도하고 비흡연자

의 간접 흡연 노출을 감소 시키고자 지방자치단체(이하 지자체)에서 금연 관련 조례제정이 2010년 8월부터 가능하게 되었으며, 이후 여러 지역사회에서 금연구역 지정 및 금연구역 내 흡연 단속과 과태료 부과와 관련하여 조례 제정 계획에 대해 지속적으로 발표하고 있다.

국외 연구에서는 실내 및 공공장소의 흡연에 대한 법적인 규제가 흡연율 및 간접흡연 노출률 감소[13,14]에 영향을 미칠 뿐만 아니라, 급성 심혈관 질환 발생을 약 13% 낮추고 [15], 호흡기 질환[16]의 발생률 감소에 긍정적인 영향을 미친다고 보고하였다. 그러나 아직까지 국내 연구 중 금연정책 실시 및 담배 접근성 제한, 금연구역 확대 등, 금연환경 조성과 관련하여 이에 대한 효과를 평가한 연구는 미비하며[17], 향후 효과적인 흡연 규제를 위해 현 수행중인 정책 효과에 대해 평가 할 필요가 있을 것으로 보인다.

따라서, 본 연구에서는 최근에 시행된 금연 관련 정책 중 지자체 금연 조례 제정의 효과를 평가하기 위해 2008년부터 2012년까지 지역사회건강조사 자료를 활용하여 흡연 관련 지표 율의 변화를 분석하고자 하였다.

대상 및 방법

지역 단위는 지역사회건강조사 자료에 포함하고 있는 법정 행정동 코드를 기준으로 시·군·구 수준으로 자료를 이용하였으며, 조사시점에 따라 행정구역 변경으로 인해 누락된 자료가 존재하였다. 지자체 조례제정이 2010년에 시행됨에 따라, 지역의 기본 특성 및 조례제정은 2010년부터 2012년까지 모두 반복적으로 조사된 251개 지역 중심으로 자료를 정리하였다. 그러나 반복측정 자료분석에서는 2008년부터 결과지표를 포함함에 따라, 행정구역 변경으로 결측치 값이 존재하는 충남 천안, 충남 연기군, 전북 전주, 경남 창원 지역을 제외하고 241개 지역을 중심으로 분석을 수행하였다.

1. 자료

지역별 조례제정 관련 자료는 자치법규정보 사이트(http://www.elis.go.kr) 에서 각 시군·구 별 조례 제정에 대한 시행일 및 과태료 부과 적용일, 최소 과태료 금액 자료를 수집하였다. 조례 시행 여부에 대해서는 시행일이 2012년 12월 31일 이전인 경우 조례 시행지역으로 정의하였으며, 그렇지 않은 경우 조례 미시행지역으로 정의하였다. 조례 시행 기간에 대해서는 2012년 12월 31일을 기준으로 시행일과의 기간을 계산하여 0-3개월/4-6개월/7-11개월/12개월 이상으로 정의하였다.

본 연구는 지역사회건강조사 사이트(https://chs.cdc.go.kr/chs/index.do)로부터 사용 가능한 2008년부터 2012년까지 조사된 자료를 활용하였다. 지역사회건강조사 자료에서 지역별 조례 시행에 따른 결과지표로 현재흡연, 매일 흡연자의 평균 흡연량 (개비), 최근 1년간 금연 시도, 금연캠페인 경험을 고려하였다. 최근 1년간 금연 시도의 경우 2010년부터 조사문항지가 변경되어, 해당 지표는 2010년 자료부터 연구에 포함하였다. 국내의 흡연율에 있어서 남녀차이가 분명함에 따라, 위의 결과지표는 남성을 대상으로 율을 산출하였으며, 지역사회건강조사 원시자료 이용지침서를 참조하여 율을 산출하였다. 또한, 지역별 조례 시행에 있어서 지역적 특성을 나타낼 수 있는 요인을 고려하기 위해, 지역사회건강조사뿐 아니라 연구보고서 및 통계청으로부터 시·군·구 단위로 산출 가능한 자료를 수집하였다.

지역적 특성 중 사회경제적 지표로 2012년 지역사회건강조사 자료에서 고졸 미만 학력 인구비율 및 비경제활동 인구비율을 고려하였다. 교육수준의 경우 선행연구를 참조하여 지역박탈지수 산출 시 고졸 미만 학력을 고려함에 따라, 동일한 기준으로 산출하였다. 비경제활동 인구는 현재 경제활동 여부 관련 문항에 대해 '아니오' 응답을 기준으로 정의하여 이용하였다.

이 밖에 지역적 특성 요인으로 시·군·구 단위의 재정자주도 및 재정자립도, 지역박탈지수를 고려하였다. 재정자주도는 지방자치단체가 자주적으로 재량권을 가지고 사용할 수 있는 재원 활용 능력을 나타내는 지표이며, 재정자립도는 지방자치단체의 재정적 자립수준을 나타내는 지표이다. 또한, 담배 구매의 접근성을 고려하기 위해 음·식료품 및 담배 소매업점 수를 포함하였다. 재정자립도[18], 재정자주도[19], 음·식료품 및 담배 소매업점 수[20] 자료는 통계청으로부터 해당 자료를 수집하였다. 지역박탈지수는 시·군·구의 1) 낙후된 주거환경 비율, 2) 노인 인구 비율, 3) 고졸 미만 학력 인구비율, 4) 가구원 기준 하위 사회계급 비율, 5) 아파트 가구 비율, 6) 자동차 미소유 가구 비율, 7) 독거 가구 비율, 8) 여성 가구주 비율을 포함하여 총 8개 지표를 종합해 산출 되는 것으로 값이 클수록 지역박탈지수가 높다고 해석한다. 해당 자료는 2005년 자료를 이용하여 산출된 값으로, 2006년부터 2009년까지 수행된 건강불평등 완화를 위한 건강증진 전략 및 사업개발 연구 보고서[21] 로 부터 수집하였다.

2. 통계분석

모든 분석에 활용된 자료는 법정 행정동 코드를 기준으로 시·군·구 단위의 율을 산출하였다. 율 산출 시 조사 시점 및 지역별 직접적 비교를 위해 2010년 추계인구의 연령별(19−29/30−39/40−49/50−59/60−69/70세 이상) 인구 비율을 고려하여 표준화율을 산출하여 분석에 이용하였다. 일부 분석에서는 성인 연령을 19−29세 이하/30−44세 이하/45−64세 이하/65세 이상으로 범주화하여 분석을 수행하였다.

2008년부터 2012년까지 조사된 현재 흡연율 추세는 회귀분석을 통해 분석하였으며, 현재 흡연율을 포함하여 결과 지표의 율의 변화에 대해 지역별 조례 시행 여부 혹은 조례 시행 기간에 효과를 평가하기 위해 반복측정 분산분석(repeated measure analysis of variance)을 이용하여 분석을 수행하였다. 반복 측정된 결과 지표에 대해 구형성 검정은

모클리검정(Mauchly test)을 이용하여 평가하였으며, 구형성 검정에 만족하지 않은 경우, *p* value 는 Huynh−Feldt−Lecoutre 교정을 이용하였다. 마지막으로, 조례 시행 여부에 따른 지역 간의 지역적 특성의 차이는 T−test 혹은 윌콕슨 순위합 검정(Wilcoxon rank sum test)을 이용하여 분석을 수행하였다. 모든 통계 분석은 양측검정에서 $p<0.05$인 경우를 통계적 유의 수준으로 판정하였고, SAS 9.3 통계패키지(SAS In−stitute Inc., Cary, NC, USA)를 사용하였다.

결과

2012년 남성 평균 흡연율은 44.8%로 2008년 이후 감소하는 경향을 보였으며(β=-0.57%/year), 매일 흡연자의 평균 흡연량(개비)도 비슷한 경향을 보였다. 금연캠페인 경험률은 2008년 이후 증가하였다가 최근 감소하였으며, 최근 1년간 금연 시도율 또한 2010년 이후 증가하다가 2012년에 감소하였다. 연령별로 연도별 차이를 보면, 30-64세 연령군의 2012년 흡연율이 2008년 및 2010년 이후 감소폭이 1%p 미만인 점을 고려했을 때, 30세 미만의 젊은 연령군의 2012년 흡연율은 2008년 대비 5.1%p, 2010년 대비 3.1%p 감소하였으며, 65세 이상 연령군은 2008년 대비 4.6%p, 2010년 대비 2.1%p 감소하였다(Table 1).

2012년 12월 31일 기준으로 지자체 금연 조례를 시행한 지역은 약 51% 수준이었으며, 이 중 41%는 1년 이상 시행한 지역이었다. 서울 및 울산 지역의 경우 2012년까지 지역구 모두 조례를 시행하였으며, 광역시 이상 지역의 조례 시행률은 약 77%로 높은 수준을 보였다. 반면 그 외 지역은 경기 지역을 제외하고 지역의 조례 시행률이 낮았으며, 특히 강원, 전남, 제주 지역이 낮았다(Figure 1, Table 2).

지역적 특성으로 천명당 음식점 및 담배소매업점 수는 제주 지역이 높았으며, 광역시 이상 지역에서는 부산 지역에서 높게 나타났다. 비경제활동 인구비율의 경우 광주 지역이 25.3%로 높았으며, 고졸 미만 교육수준 인구비율은 전북 지역이 26.3%로 가장 높게 나타났다(Table 2).

금연 조례 시행 지역과 미시행 지역간의 남성 현재 흡연율을 보았을 때, 전반적으로 조례 시행 지역의 남성 흡연율이 낮았으며, 2010년 이후부터 약 2%정도의 차이를 보였다. 조례 시행여부에 따른 지역간의 흡연율의 차이는 2009년부터 유의한 것으로 나타났으며(Table 3), 지역간의 흡연율 차이 정도에 있어서는 2008년과 비교하여 2010년부터 유의하게 차이가 있는 것으로 나타났다(Figure 2). 그러나 조례 시행 지역간의 시간에 따른 흡연율 감소 추이는 경계성 유의수준을 보였다(p_{G*T}=0.06, Table 3). 조례 시행여부에 따른 지역간의 흡연율 추이에 대해 연령별로 층화하였을 때, 30세 미만의 연령군을

제외하고 조례 시행 지역의 흡연율이 전반적으로 낮았다. 반면, 30세 미만 젊은 연령군에서 2010년부터 조례 시행지역의 흡연율이 미시행지역보다 낮아졌으며 2012년에는 그 간격이 더 커지는 것으로 나타났다(Figure 3).

흡연량의 경우, 흡연율과 동일하게 금연 조례 시행 지역이 전반적으로 낮았으나, 조례 시행 지역간의 시간에 따른 흡연량 감소 추이는 통계적으로 유의하지 않았다. 금연 시도율의 경우, 금연 조례 시행지역이 미시행지역보다 평균적으로 높았으며, 2010년이후 증가하였다가 감소하였지만, 2010년과 2011년 사이에 조례 미시행 지역(2.4%p)보다 조례 시행 지역(3.6%p)에서 더 크게 상승하였다. 캠페인 경험률은 조례 시행여부에 따른 지역간의 차이는 유의하지 않았지만(p_G =0.81), 2010년부터 2011년 사이에 조례 미시행 지역에서 2.7%p 상승한 것에 반해 조례 시행지역에서 캠페인 경험률이 5.0%p로 상승하였으며, 통계적으로 유의한 차이를 보였다(Table 3).

조례 시행 이후 기간 (조례 제정 없음/ 3개월 이내/ 6개월 이내/ 1년 이내/ 1년 이상)에 따른 지역 간의 현재 흡연율을 분석하였을 때, 12개월 이상 조례를 시행한 지역에서 남성 현재 흡연율이 2008년 이후 지속적으로 낮은 수준을 보였으며, 흡연율의 차이는 2009년부터 유의한 것으로 나타났다. 이 밖에 흡연량, 금연 시도율 및 캠페인 경험률의 경우 시행 기간에 따른 지역간의 차이는 조사 시점에 따라 차이를 보였으나, 종단적으로 분석하였을 때 유의한 차이를 보이지 않았다(Table 4).

조례 시행 여부에 따른 지역 간의 지역적 특성을 평가했을 때, 조례 시행 지역의 천명당 음식점 및 담배소매업점 수는 2.1개로 미시행지역 3.0개보다 적었으며, 고졸 미만 학력 인구비율도 17.1%로 미시행 지역 23.1%보다 낮았다. 반면 비경제활동 인구비율은 시행지역이 미시행지역보다 높은 것으로 나타났다. 또한 지역박탈지수는 미시행 지역이 높았으며, 재정자주도, 재정자립도 모두 시행 지역이 미시행지역보다 전반적으로 높았으며, 재정자립도에서 유의하게 차이를 보였다(Table 5).

고찰

본 연구에서는 국가전반에 시행되는 금연관련 정책이 아닌 지역별로 시행하고 있는 금연관련 정책에 대해 평가하고자 하였다. 연구결과, 2012년까지 50% 이상의 지역에서 조례를 시행하였으며, 전반적으로 조례 시행지역의 흡연율 및 일일 평균 흡연량은 미시행지역보다 낮았다. 또한, 조례 시행여부에 따른 지역간의 흡연율 차이는 2008년과 비교하여 2010년부터 유의하게 차이가 있는 것으로 나타났다. 특히, 최근 1년간 금연 시도율 및 금연캠페인 경험률은 2010년과 2011년 사이에 조례 시행지역이 미시행지역보다 상승 폭이 컸으며, 이는 조례 시행 기간으로 나눴을 때 담배 규제를 위한 정책이 일찍 시행된 지역의 율의 변화 크기가 컸으나, 통계적으로 유의하지 않았다.

흡연은 다양한 흡연관련 질환에 대해 예방 가능한 위해 요인이며, 흡연자뿐만 아니라 담배 연기에 노출된 비흡연자에게도 좋지 않은 건강영향을 미치는 것으로 알려져 있다. 이에 대해 국제적으로 금연 관련 정책 마련을 통해 담배 관련 건강 위험을 감소시키고 예방하기 위해 지속적으로 노력하고 있다. 일반적으로 금연을 위한 정책은 크게 가격정책과 비가격 정책으로 구분할 수 있으며, 비가격정책으로는 금연 홍보 및 금연사업 지원 등을 포함한다. 가격정책으로 인한 효과는 2004년 12월 담뱃값인상을 통해 2001년 60.9%(표준화율)였던 남성 흡연율은 2005년 51.6%, 2007년 45.0%까지 감소하였다 [22]. Shin [23]은 담배가격 인상에 따른 유도 가능한 흡연율 감소에 대해 예측한 금연의향 담배가격분석 연구에서 담배가격이 3000원이면 현재 흡연자의 약 41%, 4000원이면 약 73%가 금연할 것으로 예측한 바 있다. 일부 연구에서는 담배가격 상승률이 물가상승률과 소득상승률보다 낮아, 담배 소비로 인한 경제 부담이 크지 않음을 지적하였으며, 담뱃값 인상 시 이에 대한 고려가 필요하다고 주장하기도 하였다[5].

국내 시행된 금연관련 비가격정책으로 1995년 이후 금연구역을 지속적으로 확대하였으며, 2012년 12월 국민건강증진법 개정을 통해 공공기관 및 공중 이용시설에서 전면금연을 시행하였다. 금연 관련 정책에서 있어서 공공장소, 작업장, 음식점 등 금역구역 지정에 따른 법적 규제 효과에 대해 코크란 체계적 문헌 고찰[14]에서는 간접흡연 노출률은 감소하였으나, 현재 흡연율에 있어서는 연구마다 상이한 결과를 보였다. 유럽지역에

서 수행된 연구에서 아일랜드의 경우 2004년 3월부터 서비스업을 포함하여 작업장에서 전면적인 금연 정책을 시행하였으며, 시행 후 1년된 시점에 흡연율은 29%에서 26%로 감소하였으나, 이후 년도에 28%로 증가하였다[24]. 또한, 영국에서도 2007년 7월에 관련 법안을 시행 한 후, 1년 사이에 금연 시도율이 증가하기는 하였으나, 지속적인 효과를 보지는 못했다고 보고한 바 있다[24].

본 연구결과, 큰 폭의 변화는 아니지만 2008년 이래 흡연율은 감소하는 추세를 보였으며, 이는 30세 미만의 젊은 연령군과 65세 이상 연령군에서의 감소율에 기인한 것으로 보인다. 또한, 지역별 금연 조례 제정에 대한 효과에 있어서 조사시점 별로 분석하였을 때, 조례 제정 지역간의 효과 차이는 존재하였으나 종단적 분석을 통해 접근하였을 때 시간에 따른 흡연율 감소 폭은 조례 시행 지역간에 있어서 차이를 보이지 않았다. 이는 지역사회 건강조사 시점을 고려하여 12년 8월 이전에 금연 관련 조례를 시행한 지역과 그렇지 않은 지역으로 정의하여 분석했을 시에도, 조례 시행 지역의 흡연율이 그렇지 않은 지역보다 낮았으며, 위의 결과와 유사하게 조사시점 별로 지역간의 차이는 존재하나, 종단적 분석 시 시간에 따른 흡연율 감소 폭은 조례 시행 지역간에 있어서 차이를 보이지 않았다. 지역별 금연 조례 제정 효과에 대해 연령별로 보았을 때 30세 미만 젊은 연령군에서 2010년부터 조례 시행지역의 흡연율이 미시행지역보다 낮아지는 변화를 보였다. 젊은 연령에서의 금연 효과는 이탈리아에서 수행된 연구에서도 15-24세 연령군에서 실내 공공장소 금연 법규 시행 이후 흡연율이 크게 감소하였다고 보고한 바 있다[13]. 본 연구에서 젊은 연령의 흡연율 감소는 높은 금연 시도율과 금연캠페인 경험률이 영향을 미쳤을 것으로 보인다. 그러나 이러한 경향에 대해서는 향후 지속적인 관찰이 필요할 것으로 보인다.

대부분의 연구에서 금연 관련 정책의 대한 평가는 현재 흡연율 및 간접흡연 노출률, 흡연자의 인지 및 태도 변화 지표로 금연시도율을 고려하여 시행 전후의 율의 변화에 대해 평가하였다. 금연 정책은 일반적으로 국가 전반에 동시에 수행됨에 따라 시행 전후로 율의 변화를 평가하는 경우가 대부분 이었으며, 미국의 경우, 국가 내 많은 주(state)가 존재함에 따라 주의 금연 활동에 대해 지표를 개발하기도 하였다. 미국 금연 중재 연구(American Stop Smoking Intervention Study)에서 주의 금연 중재 정책 혹은 활동을 평

가하기 위해 주의 실내 금연 법안 규제 정도 및 담배 가격 등을 나타내는 IOI (initial outcome index) 지표와 중재 프로그램을 위한 자원, 역량, 활동 분야에 대한 SOTC (strength of tobacco control)지표를 개발하였으며[9], 두 지표 모두 주 흡연율 감소와 상관성이 있는 것으로 나타났다.

흡연은 개인을 둘러싸고 있는 환경, 사회, 문화에 따라 영향을 받을 수 있으며[12,25]. 이는 거주하는 지역[8,9], 지역의 사회경제적 수준(빈곤, 실업률)[10,11] 및 담배 구입에 있어서 접근성이 좋을수록 개인의 흡연과 관련이 있다[12]. 국내 연구에서도 2009년 지역사회건강조사 자료를 이용하여, 흡연율의 지역간 변이에 대한 관련 요인으로 지역박탈지수, 인구 천명당 주점 수, 주민 대상 금연교육 수가 관련이 있는 것으로 보고되기도 하였다[26]. 일반적으로, 지역별 조례는 금연 환경 조성을 위해 금연구역 지정 및 금연구역 내 과태료 부과에 대해 권한 부여 외에도 금연 홍보 및 캠페인 활동에 대한 내용을 담고 있다. 본 연구에서, 금연사업 실행 가능 평가를 위한 대안적 지표로 지역사회의 재정적 능력을 나타내는 재정자주도 및 재정자립도를 고려하였을 때에도, 전반적으로 조례 시행지역의 재정자주도 및 재정자립도가 미시행지역보다 높은 것으로 나타났다. 이밖에 조례 시행지역과 미시행지역간의 지역적 특성으로 사회경제학적 요소로 고려한 고졸 미만 학력 인구비율, 지역박탈지수의 경우, 조례 시행지역의 고졸 미만 학력 인구비율은 미시행 지역보다 약 6%p 정도 낮았으며, 지역박탈지수도 유의하게 낮았다. 또한, 조례 시행 지역의 천명당 음식점 및 담배소매업점 수는 2.1개로 미시행지역 3.0개 보다 낮았다. 위험을 회피하거나 극복할 수 있는 사회경제적 기회와 자원의 불평등 분포는 건강의 사회불평등을 야기 할 수 있으며[27], 지역별 금연 조례 제정에 있어서도 지역적 특성에 따라 차이를 보였다. 지역별 금연 조례 제정에 지속적으로 확대되고 있지만, 조례 제정에 있어서 과태료 부과 금액 및 금연 사업 규모 또한 상이한 부분이 있어 이에 따른 차이가 지역사회의 흡연율 감소에 어떻게 영향을 미칠지는 향후 관련 연구가 필요할 것으로 보인다.

본 연구의 경우, 지역별 조례의 실질적인 실행 정도를 평가할 수 있는 단속횟수, 과태료 부과건수, 금연 사업 예산 등 직접적인 지표를 고려하지 못하고 단순히 조례 시행만을 고려하여 실제적인 조례 효과가 분명하게 나타나지 않았을 수도 있다. 실제로 조례

내용 중 과태료 부과 시행 시점이 조례 시행 시점과 차이가 있었으며, 조례 시행 지역에서 과태료 부과 건수에 있어서도 차이가 큰 것으로 보고된 바 있다. 따라서, 금연 조례 제정에 대한 효과는 실질적인 조례 실행에 대해 평가 가능한 지표를 포함하여, 향후 장기적인 관찰을 통한 지속적인 평가가 필요할 것으로 보인다. 이 밖에, 개인을 반복적으로 추적 관찰한 자료가 아닌 생태학적 연구 접근을 통해 수행된 연구 결과로 해석 시 제한점이 존재하며, 조사문항 중 금연시도율에 있어서 2010년 조사 문항변경으로 2008년과 2009년과 조사자료를 활용하는데 어려움이 존재하였다. 또한, 연간 조사된 자료로 조례 시행 전후의 조사결과가 혼재될 수 있으며, 각 지역마다 조례 시행 시점에 차이가 있어 율 산출 시 영향을 미쳤을 것으로 보인다. 그럼에도 불구하고, 본 연구는 다년도 자료를 활용하여 금연 조례 효과에 대해 지역사회 수준에서 평가하였다는데 의미가 있으며, 향후 관련 연구를 수행하는데 기초자료로 활용될 수 있을 것으로 보인다.

결론

본 연구는 지역사회건강조사의 다년도 자료를 활용하여 국가 전반에 시행되는 법률 외에 2010년부터 시행된 지차제 금연 조례 제정의 효과에 대해 평가하고자 하였으며, 조례 시행여부에 따른 지역간의 흡연율 차이는 2008년과 비교하여 2010년부터 유의하게 차이가 있는 것으로 나타났으며, 연령별로 보았을 때 30세 미만 젊은 연령군에서 2010년부터 조례 시행지역의 흡연율이 미시행지역보다 낮아지는 변화를 보였다. 또한, 조례 시행지역과 미시행지역간의 지역적 특성에 차이를 보였다. 따라서, 지역별 정책 제정에 있어서 불평등이 야기되지 않도록 노력이 필요할 것으로 보이며, 지역별 조례제정이 확대되고 있음에 따라, 실질적인 조례 내용 실행 정도를 평가할 수 있는 지표를 포함하여 장기적인 평가가 필요할 것으로 보인다.

참고문헌

1. World Health Organization. Tobacco. 2014 [cited 2014 Oct 25]. Available from: http://www.who.int/mediacentre/factsheets/fs339/en/
2. National Cancer Institute. Secondhand Smoke and Cancer. 2011 [cited 2014 Oct 25]. http://www.cancer.gov/cancertopics/factsheet/Tobacco/ETS
3. National Cancer Institute. Harms of Smoking and Health Benefits of Quitting. 2011 [cited 2014 Oct 25]. http://www.cancer.gov/cancertopics/factsheet/Tobacco/cessation
4. Jee SH, Lee JK, Kim lS. Smoking-Attributable Mortality Among Korean Adults: 1981-2003. Korean Journal of Epidemiology 2006;28(1): 92-99. (Korean)
5. Chung YH. Directions of Fiscal Policies for Health Promotion Korea Institute for Health and Social Affairs. 2009; (Korean).
6. Ministry of Health and Welfare Affairs. Guidebook for 2014 Community-based integrated health promotion program: smoking. Seoul, Ministry of Health and Welfare Affairs. 2014; (Korean).
7. OECD. Smoking, in OECD Factbook 2013: Economic, Environmental and Social Statistics, OECD Publishing;2013 Available from:http://dx.doi.org/10.1787/factbook-2013-98-en
8. Datta GD, Subramanian SV, Colditz GA, Kawachi I, Palmer JR, Rosenberg L. Individual, neighborhood, and state-level predictors of smoking among US Black women: A multilevel analysis. Soc Sci Med 2006;63(4):1034-1044.
9. Jemal A, Thun M, Yu XQ, Hartman AM, Cokkinides V, Center MM, et al. Changes in smoking prevalence among U.S. adults by state and region: Estimates from the Tobacco Use Supplement to the Current Population Survey, 1992-2007. BMC Public Health 2011;11:512.
10. Kim CS. Yun SC. Kim HR. Khang YH. Multilevel Study on the Relationship between the Residential Distribution of High Class (Power Elites) and Smoking in Seoul. J Prev Med Public Health 2006; 39(1):30-38. (Korean)
11. Park EJ, Kim H, Kawachi I, Kim IH, Cho SI. Area deprivation, individual socioeconomic position and smoking among women in South Korea. Tob Control. 2010;19(5):383-390.
12. Chuang Y, Cubbin C, Ahn D, Winkleby M. Effects of neighbourhood socioeconomic status and convenience store concentration on individual level smoking. J Epidemiol Community Health 2005; 59(7): 568–573.

13. Gallus S, Zuccaro P, Colombo P, Apolone G, Pacifici R, Garattini S, et al. Effects of new smoking regualtions in Italy. Annals of Oncology 2006;17:346-347.
14. Callinan JE, Clarke A, Doherty K, Kelleher C. Legislative smoking bans for reducing secondhand smoke exposure, smoking prevalence and tobacco consumption. Cochrane Database Syst Rev 2010;(4):CD005992.
15. Lin H, Wang H, Wu W, Lang L, Wang Q, Tian L. The effects of smoke-free legislation on acute myocardial infarction: a systematic review and meta-analysis. BMC Public Health 2013;13:529.
16. Mackay D, Haw S, Ayres JG, Fischbacher C, Pell JP. Smoke-free legislation and hospitalizations for childhood asthma. N Engl J Med 2010;363(12):1139-1145.
17. Kim HK, Park JY, Kwon EJ, Choi SH, Cho HI. Efficacy of Smoking Cessation and Prevention Programs by Intervention Methods: A Systematic Review of Published Studies in Korean Journals during Recent 3 Years. Korean J Health Educ Promot 2013; 30(5): 61-77 (Korean).
18. Korean statistical information service. Financial independence ratio. Statistics Korea. Available from: http://www.index.go.kr/potal/main/EachDtlPageDetail.do?idx_cd=2857
19. Korean statistical information service. Fiscal self-reliance ratio. Statistics Korea. Available from: http://www.index.go.kr/potal/main/EachDtlPageDetail.do?idx_cd=2458
20. Korean statistical information service. The number of restaurant and tobacco retail store. Statistics Korea. Available from: http://kosis.kr/statHtml/statHtml.do?orgId=101&tblId=DT_1KI2002&conn_path=I3
21. Shin YJ, Kim MH, Yoon TH. Health promotion strategies and programmes development for health inequalities alleviation. Seoul: Hanyang University & Management Center for Health Promotion; 2009.
22. Korean statistical information service. Current smoking rate from Korean National Health and Nutrition Examination Survey. Statistics Korea. Available from: http://kosis.kr/statHtml/statHtml.do?orgId=117&tblId=DT_11702_N001&conn_path=I3
23. Shin KY. Analysis of 'willingness-to-quit' cigarette price [dissertation]. Seoul: Yonsei University; 2006 (Korean).

24. Nagelhout GE, de Vries H, Boudreau C, Allwright S, McNeill A, van den Putte B, et al. Comparative impact of smoke-free legislation on smoking cessation in three European countries. Eur J Public Health 2012; 22 Suppl 1:4-9.
25. Bauld L. Impact of smokefree legislation in England: Evidence review. University of Bath, 2011
26. Kim DH. Analysis of small area variation of health behavior using 2008 community health survey in Korea. Osong: Korea Centers for Disease Control and Prevention; 2010, p. 10, 47-49 (Korean).
27. People's Health Institute**.** Healthy Equity Report 2010, Tobacco Control Policy in Korea and health inequality. People's Health Institute; 2011 (Korean).

Table 1. The prevalence of smoking related factors in male according to survey year

Age groups	2008		2009		2010		2011		2012	
	LSmeans	SE	LSmeans	SE	LSmeans	SE	LSmeans	SE	LSmeans	SE
Current smoker (%)										
All	47.21	0.24	48.09	0.21	46.18	0.22	45.23	0.21	44.80	0.21
19-29	46.89	0.63	47.21	0.52	44.89	0.55	43.02	0.55	41.82	0.56
30-44	56.16	0.40	57.69	0.35	56.14	0.37	55.59	0.36	55.60	0.38
45-64	44.80	0.39	45.83	0.34	43.85	0.34	43.12	0.34	42.89	0.33
65 or	28.36	0.48	27.91	0.44	25.88	0.42	24.78	0.41	23.75	0.41
Cigarettes/day (gabi)										
All	16.38	0.04	16.56	0.02	16.47	0.01	16.49	0.01	16.17	0.01
19-29	13.56	0.09	13.58	0.04	13.61	0.01	13.83	0.03	13.58	0.01
30-44	16.68	0.06	16.75	0.03	16.51	0.01	16.46	0.02	16.20	0.01
45-64	18.28	0.07	18.76	0.03	18.67	0.01	18.58	0.02	18.18	0.01
65 or	14.76	0.12	14.71	0.05	14.88	0.00	14.97	0.03	14.66	0.03
Smoking cessation trial (%)†										
All	-	-	-	-	29.77	0.29	33.21	0.30	27.04	0.29
19-29	-	-	-	-	35.70	0.79	41.49	0.82	34.53	0.82
30-44	-	-	-	-	31.80	0.45	35.85	0.48	28.66	0.45
45-64	-	-	-	-	26.69	0.47	28.80	0.48	23.28	0.44
65 or	-	-	-	-	22.30	0.80	23.71	0.84	20.06	0.81
Experience of Anti-smoking campaign (%)										
All	82.98	0.20	82.08	0.17	83.12	0.17	87.30	0.15	81.30	0.18
19-29	84.51	0.45	84.70	0.38	84.70	0.40	87.78	0.38	81.18	0.45
30-44	86.46	0.28	85.53	0.25	86.12	0.26	89.20	0.23	83.32	0.28
45-64	81.95	0.30	80.85	0.28	82.64	0.27	87.53	0.23	81.64	0.27
65 or	72.99	0.48	70.76	0.46	72.75	0.43	80.05	0.39	74.42	0.42

LSmeans: least squares means, SE: standard error

†Because question of smoking cessation trial changed from 2010, this table included results derived from survey which conducted in 2010, 2011, 2012

Table 2. Regional characteristics in Republic of Korea

Districts	The number of community	Duration of implementation of smoke-free regulation by Dec.31 2012†					The number of restaurant and tobacco retail store/1000 person	The rate of economically inactivity population (%)	The rate of under high school education level (%)
		Not yet	0-3mo	4-6mo	7-11mo	≥12mo			
SEOUL	25	0	0	0	0	25	1.86(1.00)	22.80(2.47)	13.40(4.50)
BUSAN	16	8	2	2	1	3	3.95(3.20)	24.34(3.65)	17.32(3.88)
DAUGE	8	3	4	0	1	0	2.56(1.77)	24.06(2.38)	17.83(4.13)
INCHEON	10	2	3	5	0	0	2.79(1.98)	21.50(3.99)	17.98(3.81)
GWANGJU	5	2	1	2	0	0	2.60(1.73)	25.31(2.20)	14.8993.51)
DAEJEON	5	2	3	0	0	0	1.66(0.49)	24.79(2.92)	16.59(5.42)
ULSAN	5	0	0	0	0	5	1.88(0.62)	20.24(1.24)	16.90(1.82)
KYONGGI	44	7	5	11	9	12	1.25(0.41)	21.80(2.65)	14.98(5.08)
GANGWON	18	17	1	0	0	0	3.20(1.14)	19.71(4.77)	23.82(4.40)
CHUNGBUK	13	9	0	2	0	2	1.82(0.81)	19.68(3.49)	23.00(4.39)
CHUNGNAM	17	13	3	1	0	0	3.38(3.32)	18.48(3.98)	24.68(6.31)
JEONBUK	15	11	0	0	1	3	2.91(1.10)	20.74(4.99)	26.32(5.97)
JEONNAM	22	18	3	0	1	0	3.29(1.14)	17.67(3.34)	26.28(5.06)
GYEONGBUK	24	21	2	0	1	0	2.84(1.41)	18.86(2.88)	24.41(4.44)
GYEONGNAM	22	9	2	1	7	3	2.48(1.05)	20.44(3.61)	21.80(4.49)
JEJU	2	2	0	0	0	0	4.96(1.50)	15.14(5.91)	17.78(3.95)
Total	251	124	29	24	21	53	2.51(1.74)	20.89(3.93)	20.05(6.55)

†0-3mo: Sep.2012-Dec.2012; 4-6mo: Jun.2012-Aug.2012; 7-11mo: Jan.2012-May.2012; ≥12mo: earlier than Jan. 2012

Table 3. The effect of community level smoke-free regulation

	Survey year					p
	2008	2009	2010	2011	2012	
	LSmeans	LSmeans	LSmeans	LSmeans	LSmeans	
Current smoker (%)						p_G<.001
community without SFR	47.66	**49.79**	**48.01**	**47.31**	**47.03**	p_T<.0001
community with SFR	47.17	**48.20**	**46.00**	**45.25**	**44.91**	p_{G*T}=0.06
Cigarettes/day (gabi)						p_G<.0001
community without SFR	**16.97**	**17.19**	**17.12**	**17.05**	**16.73**	p_T<.0001
community with SFR	**16.41**	**16.56**	**16.32**	**16.43**	**16.17**	p_{G*T}=0.51
Smoking cessation trial (%)†						p_G <.0001
community without SFR	-	-	**24.44**	**26.71**	**21.86**	p_T <.0001
community with SFR	-	-	**29.79**	**33.39**	**26.98**	p_{G*T} =0.30
Experience of Anti-smoking campaign (%)						p_G =0.81
community without SFR	80.72	82.36	84.39	87.13	81.77	p_T <.0001
community with SFR	82.75	81.81	82.38	87.37‡	81.27	p_{G*T}=0.07

SFR: smoke-free regulation, LSmeans: least squares means

p_G :p value for group effect; p_T:p value for time effect; p_{G*T} :p value for interaction effect between group and time

Communities classified into two groups according to implementation of smoke-free regulation by Dec.31 2012

Repeated measure analysis of variance model is used for estimation. It model include group (community with or without smoke-free regulation), time, and interaction between group and time.

†Because question of smoking cessation trial changed from 2010, this table included results derived from survey which conducted in 2010, 2011, 2012

‡The rate differences between 2010 and 2011 by community with or without smoke-free regulation was statistically significant (p=0.02)

Bold value indicates p value <0.05 for cross-sectional effect

Table 4. The effect of duration of implementation of smoke-free regulation†

	Survey year					p
	2008	2009	2010	2011	2012	
	LSmeans	LSmeans	LSmeans	LSmeans	LSmeans	
Current smoker (%)						
Not yet	47.66	**49.79**	**48.01**	**47.31**	**47.03**	p_G =002
0-3mo	48.21	**48.16**	**46.95**	**46.18**	**45.74**	p_T <.0001
4-6mo	46.93	**48.69**	**46.69**	**45.77**	**45.18**	p_{G*T}=0.47
7-11mo	47.68	**50.47**	**46.80**	**46.57**	**46.57**	
≥12mo	46.60	**47.31**	**44.96**	**44.14**	**43.86**	
Cigarettes/day (gabi)						
Not yet	**16.97**	**17.19**	**17.12**	**17.05**	**16.73**	p_G <.0001
0-3mo	**16.52**	**16.56**	**16.29**	**16.57**	**16.16**	p_T =0.002
4-6mo	**16.54**	**16.55**	**16.37**	**16.49**	**16.16**	p_{G*T}=0.84
7-11mo	**16.55**	**17.19**	**16.65**	**16.62**	**16.82**	
≥12mo	**16.26**	**16.37**	**16.21**	**16.28**	**15.97**	
Smoking cessation trial (%)‡						
Not yet	-	-	**24.44**	**26.71**	**21.86**	p_G <.0001
0-3mo	-	-	**29.23**	**30.89**	**24.95**	p_G <.0001
4-6mo	-	-	**30.93**	**32.09**	**28.85**	p_{G*T}=0.20
7-11mo	-	-	**26.59**	**31.04**	**24.52**	
≥12mo	-	-	**30.53**	**36.02**	**27.90**	
Experience of Anti-smoking campaign (%)						
Not yet	80.72	82.36	**84.39**	87.13	81.77	p_G =0.40
0-3mo	80.36	82.04	**83.64**	87.55	81.58	p_T <.0001
4-6mo	84.66	83.44	**84.69**	89.34	82.32	p_{G*T}=0.15
7-11mo	80.20	78.42	**82.96**	87.89	80.27	
≥12mo	83.88	81.97	**80.52**	86.23	80.95	

SFR: smoke-free regulation, LSmeans: least squares means

p_G :*p* value for group effect; p_T :*p* value for time effect; p_{G*T} :*p* value for interaction effect between group and time

Repeated measure analysis of variance model is used for estimation. It model includes group (not yet/0-3mo/4-6mo/7-11mo/≥12mo), time, and interaction between group and time.

Duration calculated from the enforcement date of the smoke-free regulation to Dec.31 2012 and then communities classified into four groups (not yet/0-3mo/4-6mo/7-11mo/≥12mo).

†0-3mo: Sep.2012-Dec.2012; 4-6mo: Jun.2012-Aug.2012; 7-11mo: Jan.2012-May.2012; ≥12mo: earlier than Jan. 2012

‡Because question of smoking cessation trial changed from 2010, this table included results derived from survey which conducted in 2010, 2011, 2012

Bold value indicates *p* value <0.05 for cross-sectional effect

Table 5. The differences of regional characteristics by smoke-free regulation

	Community without SFR	Community with SFR	*p*
The rate of economically inactivity population (%)	19.97(4.46)	21.79(3.08)	<0.001
The number of restaurant and tobacco retail store/1000 person	2.98(2.00)	2.06(1.28)	<0.0001
The rate of less than a high school education level (%)	23.10(5.48)	17.08(6.14)	<0.0001
Regional deprivation index	0.44(0.82)	-0.46(0.72)	<0.0001
Fiscal self-reliance ratio (%)			
at 2008	17.50(12.90-25.10)	39.10(23.50-55.20)	<0.0001
at 2009	18.05(12.65-24.80)	38.40(23.80-55.20)	<0.0001
at 2010	18.25(12.45-25.70)	38.60(23.10-54.40)	<0.0001
at 2011	17.40(12.50-24.90)	40.50(26.40-50.60)	<0.0001
at 2012	16.95(12.30-26.05)	39.00(25.50-49.10)	<0.0001
Financial independence ratio (%)			
at 2008	65.95(61.30-70.65)	70.70(61.70-76.90)	<0.001
at 2009	65.30(60.90-69.35)	69.70(61.30-76.70)	<0.001
at 2010	63.00(58.30-67.45)	67.50(58.80-73.90)	<0.001
at 2011	63.40(58.85-67.50)	66.90(59.20-72.60)	<0.01
at 2012	64.20(59.25-67.35)	66.40(57.80-72.10)	0.01

SFR: smoke-free regulation

P value obtained from T-test for parametric test and Wilcoxon rank sum test for non-parametric test.

Result presents mean with standard deviation or median with interquartile range for non-normal distribution.

Figure 1.

The communities with implementation of smoke-free regulation by Dec.31 2012

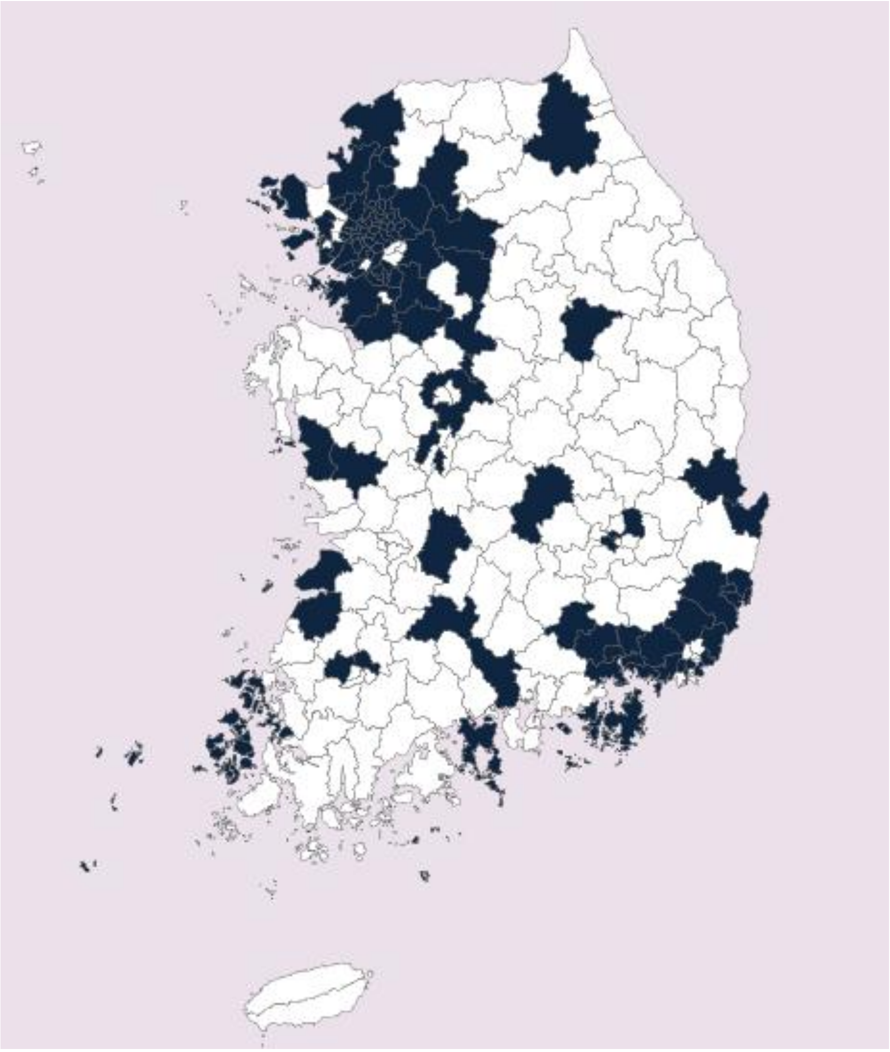

Black indicates the communities with implementation of smoke-free regulation by Dec.31 2012.

Figure 2. The effect of community level smoke-free regulation on current male smoking

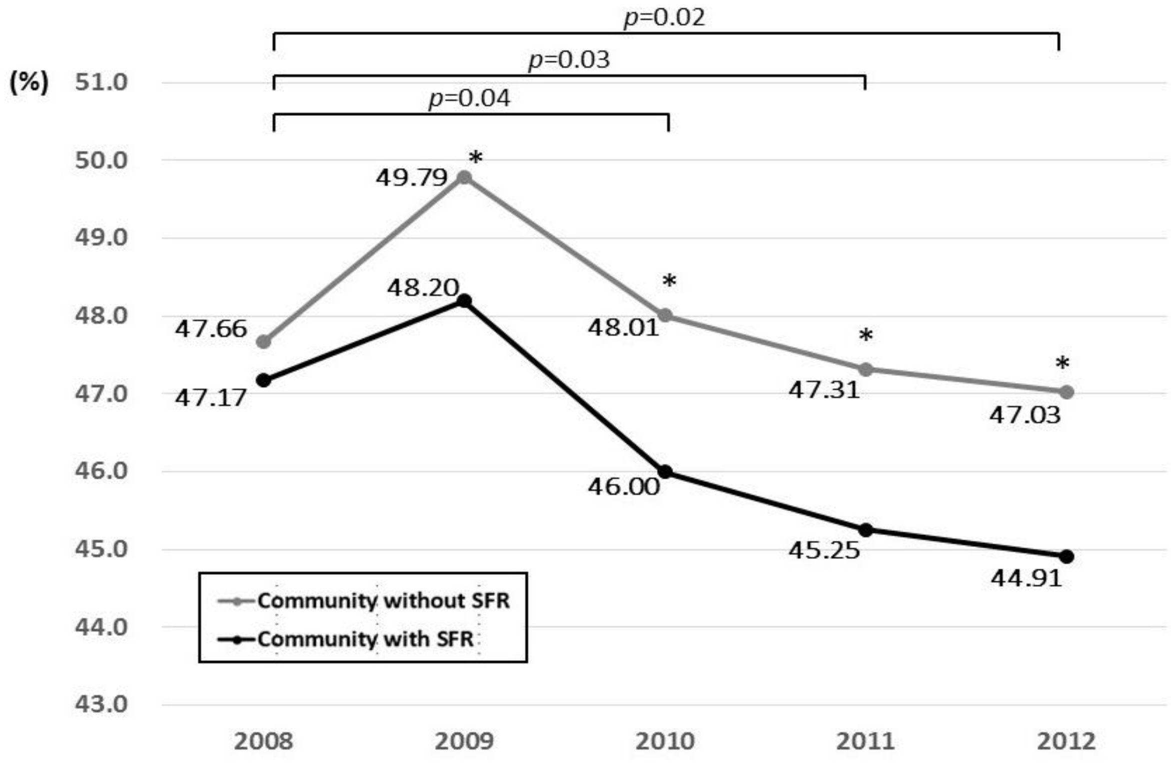

SFR: smoke-free regulation

Black dot indicates community with smoke-free regulation and gray dot indicates community without smoke-free regulation. It results obtained from repeated measure analysis of variance model, which includes group (community with or without smoke-free regulation), time, and interaction between group and time. The *p* value indicates the group difference at survey year compared to baseline survey and * indicates *p* value <0.05 for group difference at survey year.

Figure 3. The effect of community level smoke-free regulation on current male smoking by age groups

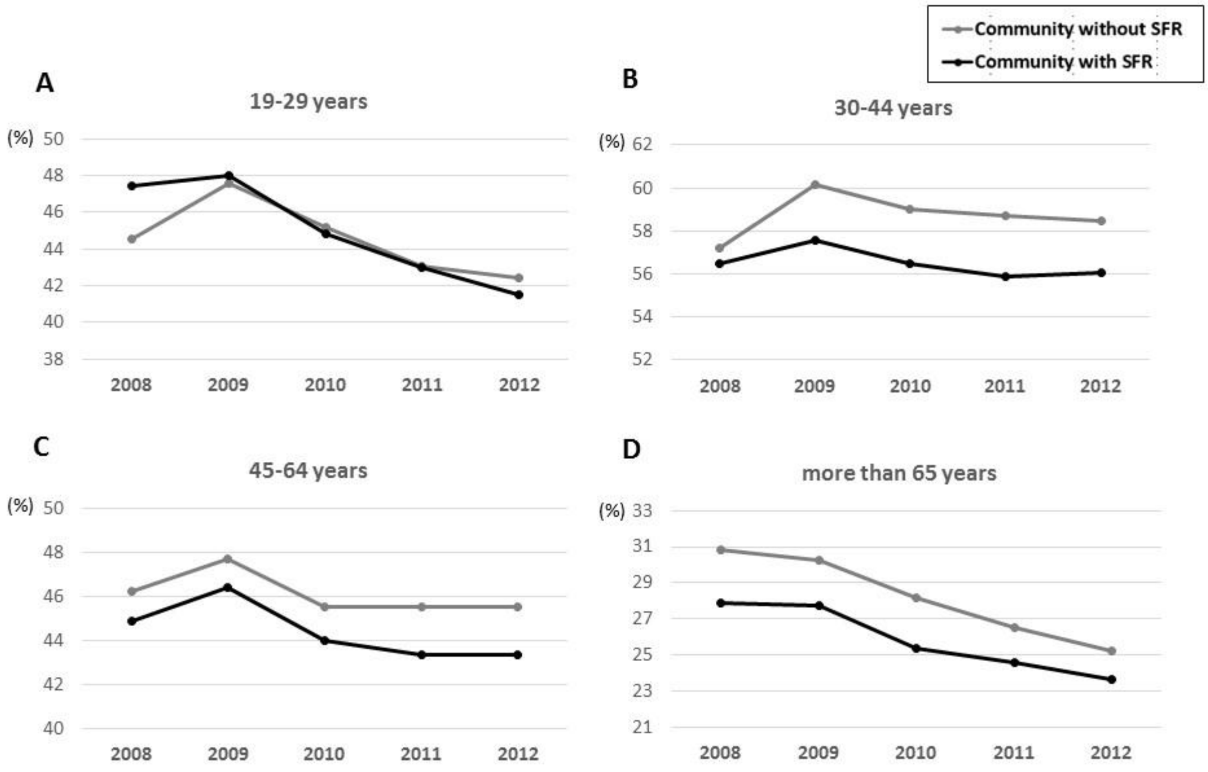

SFR: smoke-free regulation

Black dot indicates community with smoke-free regulation and gray dot indicates community without smoke-free regulation. It results obtained from repeated measure analysis of variance model, which includes group (community with or without smoke-free regulation), age group (19-29yrs/30-44yrs/45-64yrs/more than 65yrs), time, and interaction between them.
